# A pilot program to improve TB care with primary and specialty care coordination in TB public health clinic

**DOI:** 10.5588/ijtldopen.24.0302

**Published:** 2024-09-01

**Authors:** P. Jadhav, G. Boudoin, R. Miles, S. Seymour, J. Parham, A. Wolfe, J. Ali

**Affiliations:** ^1^ Louisiana State University Health Sciences Center, Department of Medicine, Section of Pulmonary/Critical Care, Allergy and Immunology, New Orleans, LA, USA;; ^2^ Wetmore TB Clinic, New Orleans, LA, USA;; ^3^ Office of Public Health, Region 1, Louisiana Department of Health, LA, USA.

**Keywords:** tuberculosis, comorbidities, primary care, public health

## Abstract

**BACKGROUND:**

The Wetmore Tuberculosis (TB) Clinic in New Orleans serves patients who often lack primary care (PC) or specialty care (SC), which is complicated by comorbidities. An initiative to provide on-site PC and coordinate care aims to enhance TB patient management.

**METHODS:**

Data collection involved categorizing patients based on their PC status: Group I (regular PC), Group II (intermittent PC), and Group III (no PC), with on-site Nurse Practitioner-based Bridge Care (NPBC) provided as needed.

**RESULTS:**

Over 12 months, 209 out of 354 patients required NPBC and PC/SC coordination, with a 20% shift from Group III to Group I, reducing the need for NPBC.

**CONCLUSION:**

The program improved TB care at Wetmore TB Clinic, offering a potential model for other TB clinics to enhance patient adherence and TB and post-TB treatment follow-up.

## RÉSUMÉ

**CONTEXTE:**

La clinique de TB de Wetmore à la Nouvelle-Orléans, États Unis, dessert des patients qui manquent souvent de soins primaires (PC, pour l’anglais « *primary care* ») ou de soins spécialisés (SC, pour l’anglais « *specialty care* »), ce qui est compliqué par des comorbidités. Une initiative visant à fournir des ordinateurs sur place et à coordonner les soins vise à améliorer la prise en charge des patients atteints de TB.

**MÉTHODES:**

La collecte des données a consisté à catégoriser les patients en fonction de leur statut de PC : Groupe I (PC régulier), Groupe II (PC intermittent) et GROUPE III (pas de PC), avec des soins de transition basés sur l'infirmière praticienne (NPBC, pour l’anglais « *Nurse Practitioner-based Bridge Care* ») sur place fournis au besoin.

**RÉSULTATS:**

Sur une période de 12 mois, 209 patients sur 354 ont nécessité une coordination NPBC et PC/SC, avec un passage de 20% du groupe III au groupe I, réduisant ainsi le besoin de NPBC.

**CONCLUSION:**

Le programme a amélioré les soins contre la TB à la clinique de TB de Wetmore, proposant ainsi un modèle potentiel pour d'autres cliniques afin d'améliorer l'observance des patients et le suivi du traitement de la TB et de la période post-traitement.

For over 50 years, the Wetmore TB Clinic in Region 1, New Orleans, has been providing TB care to residents. The clinic receives support from adult and pediatric pulmonary and infectious disease faculty from Louisiana State University Health Science Center (LSUHSC; New Orleans, LA, USA) and Tulane University Medical School (TUMC; Tulane, LA, USA). It also serves as a teaching site for public health students, residents, and fellows from these institutions, offering unique experiences in outpatient TB evaluation and management.

Many patients at the Wetmore TB Clinic lack designated primary care (PC) or specialty care (SC), are uninsured or underinsured, and face healthcare inequities and disparities.^1^ Additionally, they often have multiple high-risk comorbidities, which can lead to poor TB treatment outcomes and hinder achieving a cure.^2,3^ Like many other TB outpatient units in the United States and elsewhere, the Louisiana Department of Health mandates the clinic to evaluate patients for active or latent TB and provide medical and nursing follow-up focused solely on TB management. However, managing comorbidities and care coordination within a TB clinic setting is challenging and often fragmented.^4,5^ Therefore, it is crucial to identify common comorbidities in TB patients early and on-site to ensure sustained co-management.^6^ This need prompted the creation of a pilot model at the Wetmore TB Clinic with the following objective: to develop a structured protocol for comprehensive care for all Wetmore TB Clinic patients, including 1) bridge primary care; 2) establishing pathways for PC and SC follow-up, regardless of insurance status; 3) reducing healthcare disparities and inequities.

## METHODS

The protocol for this process improvement pathway included the following: 1) data collection and study population: from November 2022 to November 2023, 354 subjects attending the Wetmore TB Clinic were interviewed and evaluated for their TB status, including i) active TB, ii) latent TB, iii) TB under investigation, iv) TB follow-up. 2) Evaluation of TB-associated comorbidities: patients were screened for comorbidities such as diabetes, hypertension, chronic obstructive pulmonary disease (COPD), and HIV. 3) PC status evaluation: an on-site Nurse Practitioner (NP), under the collaborative supervision of the Medical Director, categorized patients into three groups: i) Group I: regular PC; ii) Group II: intermittent PC without regular follow-up; iii) Group III: no established PC. 4) Provision of bridge care: The NP assessed and managed comorbidities by providing on-site Nurse Practitioner based Bridge Care (NPBC) where necessary. 5) Identification of PC and SC coordination needs: processes were established for appropriate referrals and future appointments through structured mechanisms.

## RESULTS AND EVALUATION

This pilot program was evaluated at 4, 8, and 12 months:

1) Data verification: demographic and clinical data for 354 subjects revealed that 56 (15.8%) had active TB, 134 (37.09%) had latent TB, 40 (11.3%) had no disease/infection, and 124 (35%) were under TB evaluation. Comorbidities were identified in 131 (37%) patients with hypertension, 24 (6.8%) with HIV, 47 (13.3%) with diabetes, and 152 (42.9%) with other comorbidities.
2) PC and SC needs and action plan: by November 2023, 209 (59%) of the 354 patients required NPBC and PC/SC navigation. The NP provided counselling and assistance to 58 (16%), immediate bridge care to 83 (24%), and referrals for 78 (22%) with comorbidities. Specific needs addressed included hypertension, diabetes, COPD/asthma, gastrointestinal issues, mental health, cardiology, substance use, musculoskeletal/nerve issues, HIV follow-up, and smoking cessation.
3) Actions taken to address barriers related to transportation, language, pharmacy access, and medication costs led to a significant outcome. These measures resulted in a 40% reduction in appointment no-show rates.
4) The PCP group categorization and conversion study initially evaluated patients between November 2022 and March 2023. This evaluation resulted in the categorization of 110 patients into three groups: Group I, comprising 39 patients (35%), Group II, consisting of 44 patients (40%), and Group III, including 27 patients (25%). A mid-evaluation conducted eight months later indicated a shift in the group distributions, with Group I increasing to 58 patients (45.0%), Group II decreasing to 31 patients (24.0%), and Group III rising to 40 patients (31%). At the final evaluation, conducted 12 months after the initial assessment, the patient distribution was as follows: Group I had grown significantly to 113 patients (54.1%), Group II included 34 patients (30.9%), and Group III encompassed 57 patients (27%). Notably, there was a 20% increase in the number of patients transitioning from no primary care (PC) to regular primary care (PC), which consequently reduced the need for on-site nurse practitioner-based care (NPBC) during subsequent visits.

## DISCUSSION

TB can be cured with effective, uninterrupted treatment, but treatment adherence is challenging, especially when initial treatment and follow-up are associated with additional challenges such as comorbidities, homelessness, imprisonment, substance use, and mental health issues.^7^

Community-based PC coordination can improve TB care and reduce disparities. The COVID-19 pandemic has highlighted the need for a comprehensive care model beyond TB treatment alone.^8–10^ Strategies to improve adherence include patient-centered care, comprehensive case management, patient education, and engagement in management decisions. Building trust with healthcare workers and addressing barriers to adherence is crucial for successful TB management.

Material incentives may have short-term benefits for clinic attendance but are insufficient for long-term adherence without a comprehensive care model involving the patient, PC provider, and TB team.^11^ Community-based primary health care can significantly reduce TB morbidity and mortality, particularly in vulnerable populations.^12,13^

This process improvement program at the Wetmore TB Clinic demonstrated initial success in integrating on-site PC into a comprehensive TB care model, with positive outcomes. The model shows promise for improving TB treatment outcomes, reducing recurrence rates, and managing long-term post-TB lung disease (PTLD).

## CONCLUSION

TB care and follow-up at the Wetmore TB Clinic improved by addressing on-site PC/SC needs, eliminating barriers to care, and focusing on SC pathways for comorbidities. This model can serve as a template for other communities and regional and national TB clinics to establish pathways of PC and SC, focusing on underserved and underinsured patients, thereby reducing health inequities and disparities.
